# Pontic site development for fixed dental prostheses with and without soft tissue grafting: 1-year results of a cohort study

**DOI:** 10.1007/s00784-022-04582-y

**Published:** 2022-07-01

**Authors:** Franz J. Strauss, Brandon J. Huber, Ana Valdés, Ronald E. Jung, Sven Mühlemann, Daniel S. Thoma

**Affiliations:** 1 Clinic of Reconstructive Dentistry, Center of Dental Medicine, University of Zurich, Plattenstrasse 11, CH-8032 Zurich, Switzerland; 2 Department of Conservative Dentistry, Faculty of Dentistry, University of Chile, Santiago, Chile; 3 Departamento de Rehabilitación Cráneo Facial Integral, Universidad de Los Andes, Santiago, Chile

**Keywords:** Soft tissue augmentation, Soft tissue volume, Subepithelial connective tissue graft, Fixed dental prosthesis, Pontic site

## Abstract

**Aim:**

To describe and compare the pontic site development for fixed-dental prostheses (FDPs) with and without soft tissue grafting up to one-year post insertion of FDPs.

**Materials and methods:**

A convenience sample of 24 patients participating in an ongoing RCT was provided with three-unit tooth-borne FDPs. Six patients received a subepithelial connective tissue graft (SCTG) at the pontic site, whereas 18 patients were treated without any soft tissue graft (CONTROL). Digital impressions were taken prior to tooth preparation, after tooth preparation, after insertion of the final FDP, and at the 1 year of follow-up. The obtained stereolithography files (STL) were superimposed and profilometric as well as linear changes of the soft tissue profile were assessed at the pontic regions. Profilometric outcomes included changes of the ridge contour, the alveolar ridge width, and the crown height of the pontic. Further outcomes assessed included: the papilla index, the pink esthetic score (PES), probing depth (PD), bleeding on probing (BOP), and plaque control record (PCR). Descriptive and nonparametric statistics were applied for all outcome measures.

**Results:**

The median profilometric contour between tooth preparation and 1 year after the insertion of the final FDP decreased by − 0.25 mm [Q1, Q3: − 0.36, 0.14] in the CONTROL group and increased by 0.61 mm [Q1, Q3: − 0.18, 1.06] in the SCTG group (intergroup *p* = 0.038). The alveolar ridge width between prior to tooth preparation and the one-year follow-up amounted to − 0.12 mm [Q1, Q3: − 0.74, 0.70] (= loss) in the CONTROL group and to 2.23 mm [Q1, Q3: 0.62, 3.86] (= gain) in the SCTG group (intergroup *p* = 0.032). At one year, the median crown height of the pontic tended to decrease by − 1.24 mm [Q1, Q3: − 2.05, − 1.05] in the SCTG group (intragroup *p* = 0.094) and by − 0.22 mm [Q1, Q3: − 0.58, 0.66] in the CONTROL group (intragroup *p* = 0.831), with significant differences between the groups (intergroup *p* = 0.022). The papilla index between prior to tooth preparation and one year of follow-up improved significantly in both groups (*p* < 0.05). Between FDP delivery and one year of follow-up, the PES values decreased significantly in the CONTROL group (intragroup *p* = 0.007), while in the SCTG group the change was not significant (intragroup *p* = 0.875). Clinical parameters (PD, BOP, and PCR) remained stable over time and did not differ between the groups at any time point (intergroup *p* > 0.05).

**Conclusion:**

Within the limitations of the present study, soft tissue grafting tends to limit contour changes at pontic sites, thus maintaining the esthetic outcomes over time. The lack of soft tissue grafting results in stable clinical outcomes; however, it may lead to a decrease in aesthetic outcomes over time.

**Clinical relevance:**

Autogenous soft tissue grafting seems to be a valid therapeutic option for the development of the pontic site to restore ridge defects prior to the delivery of fixed dental prostheses and to limit dimensional changes over time.

**Supplementary Information:**

The online version contains supplementary material available at 10.1007/s00784-022-04582-y.

## Introduction

A missing tooth in the buccal segment can lead to diverse consequences like decreased masticatory function, antagonist elongation, and dental tipping. Such a tooth gap can be replaced in partially edentulous patients with a fixed dental prosthesis (FDP).

FDPs are a common therapeutic treatment with a large body of clinical evidence showing high success and survival rates [1–3]. There are diverse pontic designs of the FDP, whereby the ovate pontic design results in the most esthetic soft tissue outcomes for the prosthetic tooth [4]. For the establishment of such an ovate pontic and an esthetic soft tissue outcome, the use of a provisional FDP for soft tissue conditioning is necessary [5]. The provisional FDP allows to imitate the papilla and emergence profile, thereby changing the soft tissue in the tooth space from a flat to a scalloped shape. This soft tissue conditioning is possible due to the ability of adding material below the pontic of the provisional FDP, but in certain clinical situations, a deficiency in soft tissues hinders a correct soft tissue conditioning. To remedy this situation, there is the possibility to augment the soft tissue or volume deficiency using a subepithelial connective tissue graft (SCTG) originated from the palate [6].

Thus far, SCTGs are considered as the gold standard for soft tissue volume augmentation around teeth, implants, or partially edentulous sites [7]. Notwithstanding, their performance at pontic sites has not been thoroughly investigated likely due to the difficulties to assess the soft changes in those regions. However, with the introduction of three-dimensional digital images formatted as Standard Tessellation Language (STL) files, these limitations have been overcome by allowing a superimposition of digital impressions from various time points. Consequently, this method enables to analyze the soft tissue changes over time [8]. In fact, several in vitro, preclinical, and clinical studies confirmed that this workflow is suitable for analyzing profilometric and volumetric changes of soft tissues over time [8]. Surprisingly and although there are some mid-term and few long-term studies reporting on soft tissue changes at pontic sites with or without soft tissue grafting [8, 9], the available clinical data are rather limited.

The aim of the present study was, therefore, to describe and compare the pontic site development with and without soft tissue grafting up to 1-year post insertion of fixed dental prostheses (FDPs).

## Material and methods

### Study design and study groups

The present study was designed as a prospective observational trial involving patients of an ongoing randomized controlled clinical study performed at the Clinic of Reconstructive Dentistry, Center of Dental Medicine, University of Zurich, Switzerland, and Facultad de Odontología, Universidad de los Andes, Santiago, Chile. The study had been approved by the local ethics committee KEK-ZH-Nr 2015–0641, and all patients signed an informed consent prior to inclusion. In brief, all patients received a three-unit tooth-borne FDP in the molar region of the upper or lower jaw. Details of the study design can be found elsewhere (German Clinical Trials Register; DRKS00010423). Out of this pool of patients, 24 patients were selected in order of appearance according to their convenient accessibility applying a non-probabilistic sampling method [10] and based on the following inclusion criteria:

- Available digital scans prior to tooth preparation, after tooth preparation, after insertion of the final FDP, and at 1 year
- Sufficient quality of the scans to allow superimposition at the pontic region
- Pontic site with (SCTG) or without soft tissue grafting (CONTROL)

**Fig. 1:**
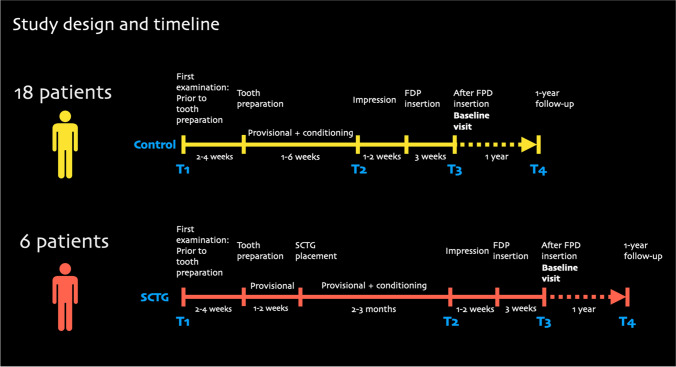
Study design and timeline

The study design and timeline are depicted in Fig. 1. This manuscript was prepared in accordance with the STROBE statement for improving the quality of observational reports (https:// www.equator-network.org/reporting-guidelines/strobe/).

### Prosthetic and surgical procedures

Tooth preparation of the mesial and distal abutment teeth was conducted following the standards of the Clinic of Reconstructive Dentistry, University of Zurich. Patients were subsequently provided with a three-unit provisional prosthesis. Whenever the pontic area presented a contour deficit, a soft tissue grafting procedure was recommended. If the patients agreed on such a surgical intervention, the pontic site was augmented using a subepithelial connective tissue graft (SCTG). In brief, sulcular incisions around the neighboring teeth were made and connected with a horizontal incision over the crest of the edentulous alveolar ridge. Subsequently, a full-thickness flap was elevated in the crestal area, followed by the preparation of a split-thickness flap on the buccal side (pouch preparation). Then, a SCTG was harvested from the palate using a single-incision technique [11] and placed in the recipient pouch in the desired position (buccally and crestally). The graft was fixed with a horizontal mattress sutured to the lingual flap. The primary flap was repositioned, and wound closure was secured with 5.0 interrupted single sutures. A representative case of the surgical procedure is presented in Supplementary Fig. 1. The provisional FDP was then adapted to prevent any undue pressure on the augmented pontic site. Sutures were removed 7–10 days later. Pontic site conditioning started 2–4 weeks after soft tissue augmentation by adapting the provisional FDP. In case patients were unwilling to undergo a surgical intervention (SCTG) or if the pontic site did not present a contour deficit, pontic site conditioning was initiated immediately after the tooth preparation and insertion of the provisional FDP.

Pontic site conditioning was performed by extending the PMMA-based provisional FDP with added composite beneath the pontic of the FDP. Thereby, the pontic of the provisional FDP was changed from its original shape to a convex ovate pontic leading to some pressure on the pontic site and the underlying soft tissue. This procedure was performed individually one to three times over a 1- to 6-week period. In other words, the longest conditioning period lasted 6 weeks, and the final impression was taken immediately during the last visit. In a subsequent visit, the final FDP was delivered.

### Image acquisition and matching of stereolithographic models

Most digital impressions of the study sites were taken by an optical scanner (3Shape, Copenhagen, Denmark). Some impressions were taken conventionally followed by scanning the casts with a laboratory scanner (Imetric 3D, Courgenay, Switzerland). The examined time points were: prior to tooth preparation (T1), after tooth preparation/insertion of the provisional (T2), after insertion of the final FDP (or baseline visit) (T3), and 1-year later (T4). The resulting stereolithographic (STL) files were imported into an image analysis software (SWISSMEDA Software; Swissmeda AG, Zurich, Switzerland). The initial STL file was individually superimposed with three STL-files that emerged from the other time points resulting in three superimpositions.

### Outcome measures

#### Clinical parameters

Clinical and periodontal measurements included: probing depth (PD), bleeding on probing (BOP), and the plaque control record (PCR).

#### Aesthetic parameters

The Jemt papilla index (JEMT) [12] and the pink esthetic score (PES) [13] were calculated using clinical photographs taken at the different time points.

#### Profilometric measurements

**Fig. 2:**
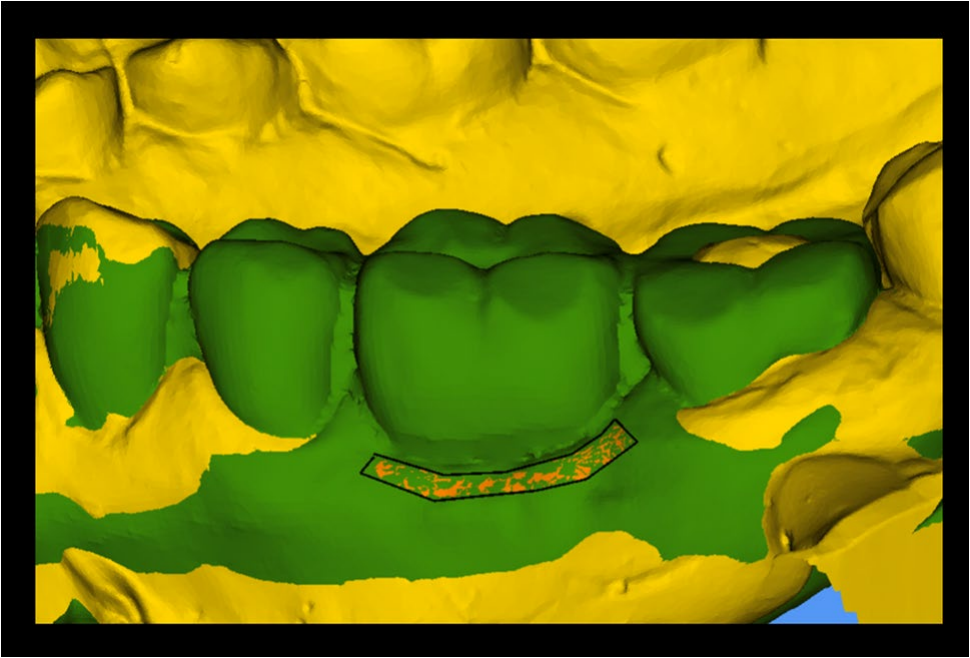
Region of interest (ROI) for the profilometric measurements. The ROI is outlined in black. Superimposition of STL model (yellow) prior to tooth preparation and one‐year follow‐up (green)

In order to assess the contour changes between the superimpositions (time points), a region of interest (ROI) was defined at the buccal aspect of the pontic site. The ROI was defined by the following borders: mesial to distal line angle of the pontic site (width) and 1 mm below the mid-facial mucosal margin to 3 mm apically (height) (Fig. 2). The software program calculated the mean distance (MD) between the surfaces (time points). This resulted in three different superimpositions:

- T1–T2: Prior to tooth preparation and post tooth preparation/insertion of the provisional FDP
- T1–T3: Prior to tooth preparation and after insertion of the final FDP or baseline
- T1–T4: Prior to tooth preparation and one-year of follow-up
- T3–T4: After insertion of the final FDP or baseline and one-year follow-up

#### Linear measurements: crown height

**Fig. 3:**
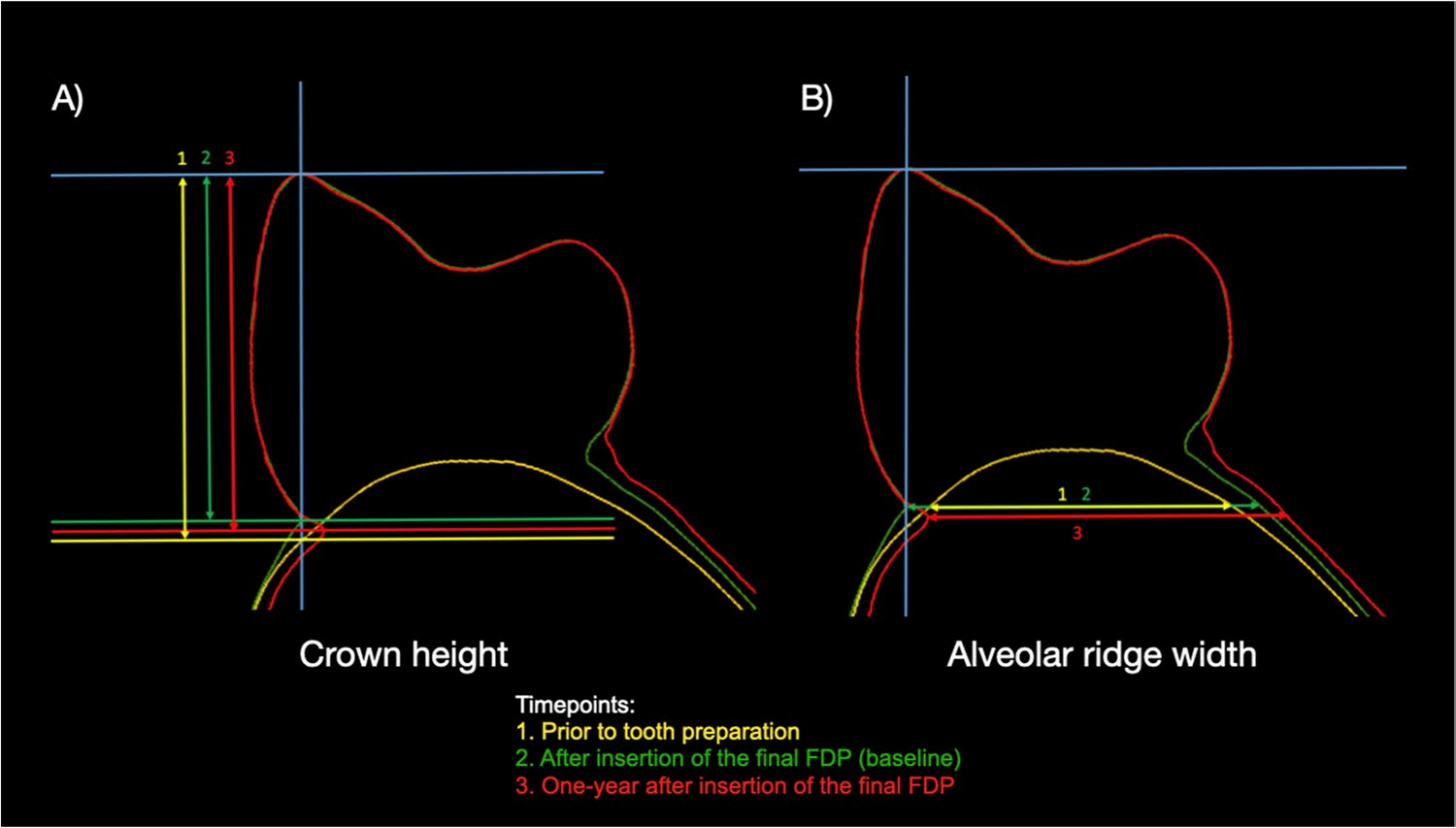
Linear measurements of the crown height of the pontic (**A**) and the alveolar ridge width (**B**) at the different time points. Abbreviations: FDP, fixed dental prosthesis

To assess the crown height (CH) and the changes over time, linear measurements were performed on cross sections of the obtained and superimposed surface scans at the different time points. The pontic of the FDP was divided into two similar segments with a longitudinal slice coinciding with the tooth axis (Fig. 3A). Thereby, it was possible to measure the crown height of the pontic by measuring the distance between the buccal crown cusp and the mucosal margin. The crown height of the pontic was measured at baseline (after the insertion of the final FDP) and at one-year post insertion of the final FDP. Finally, and in order to investigate the early changes at the pontic sites, the intraoral scan data at crown delivery was merged, aligned, and superimposed with the initial situation (prior to tooth preparation) using a virtual pontic.

#### Linear measurements: alveolar ridge width

To assess the horizontal alveolar ridge width (ARW) and the changes over time, linear measurements were performed on cross sections of the obtained and superimposed surface scans at the different time points. The ARW was measured by calculating the distance of a line perpendicular to the tooth axis connecting the gingival/pontic margin to the lingual/palatal contour (Fig. 3B). This distance was measured at all time points, and the changes were subsequently calculated.

#### Intra-reliability

The intra-examiner reproducibility of the crown height and the alveolar ridge width measurements were tested by the intra-class correlation coefficient (ICC) test. A blinded and calibrated clinician who was not involved in the surgical or prosthetic treatment performed all measurements. Both crown height (CH) and alveolar ridge width (ARW) were determined on two different occasions at least 1 month apart. For the 2nd occasion, 10 cases were randomly selected using a computer-generated list (www.randomizer.org). For the crown height, the ICC amounted to 0.997 (CI: 0.96–0.99) and for ARW to 0.996 (CI: 0.98–0.99) indicating an excellent intra-reliability.

### Statistical analysis

A software program (Excel, Microsoft Corporation, Redmond, WA, USA) was used to process the data. For the metric variables, mean, standard deviations, median, and quartiles were calculated. The comparisons of the group medians of the metric variables were performed with nonparametric methods (Wilcoxon-Mann–Whitney (WMW) test) because of the small sample sizes and non-normality of the data. Changes over time were analyzed nonparametric with the Wilcoxon signed-rank test for each group. All statistical analyses were conducted using Prism v9 (GraphPad, La Jolla, CA, USA).

### Results

**Fig. 4:**
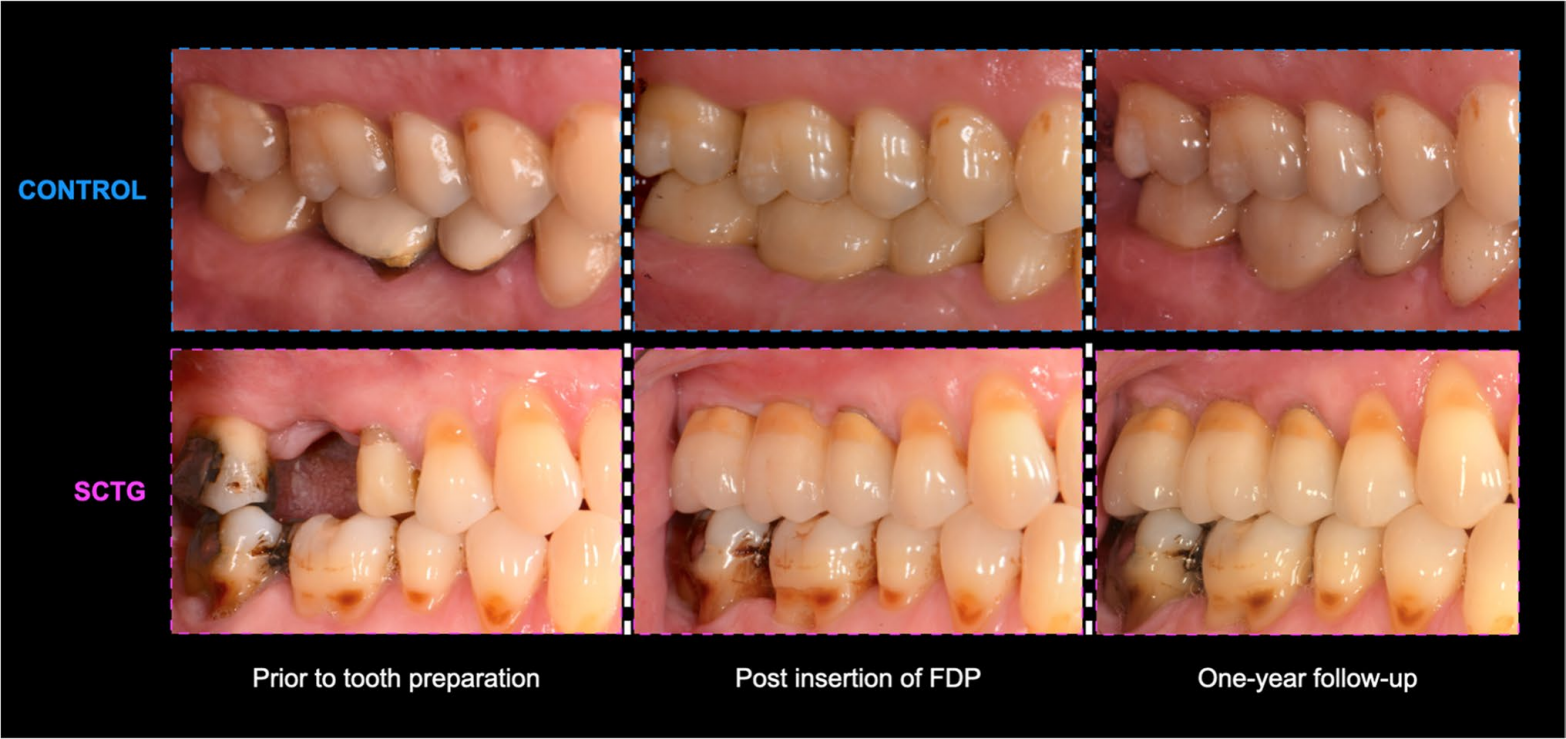
Representative cases of each treatment modality at different time points

A total of 24 participants were included in the analysis, 18 from CONTROL group and 6 from the SCTG group. The treatment time from tooth preparation to FDP insertion differed between the groups: 3–12 weeks (CONTROL) and 6–24 weeks (SCTG). Two representative cases of each treatment modality are displayed in Fig. 4.

### Clinical parameters

Clinical parameters, namely, PD, BOP, and PCR, at the different time points are shown in Table 1. The median PD change between insertion of the final FDP (baseline) and 1 year of follow-up amounted to 0.17 mm in the CONTROL group and 0.12 mm in the SCTG group without significant changes over time in any group (*p* > 0.05). The median BOP did not change significantly over time in neither of the groups (Table 1). With respect to the plaque levels, the median change between baseline and 1 year of follow-up was 0 in both groups (Table 1).

**Table 1:** Clinical outcomes for CONTROL and SCTG group prior to tooth preparation (T1), after insertion of the fixed dental prosthesis (T3), and at one-year follow-up (T4) including the changes between different time points. Patient-level analysis with means, standard deviations (SD), medians, interquartile ranges (IQR), range from minimum to maximum for both groups (CONTROL and SCTG)

		*n*	CONTROL						*n*	SCTG						
			Mean ± SD	Q1	Median	Q3	Range min to max	Paired *p *value		Mean ± SD	Q1	Median	Q3	Range min to max	Paired *p* value	Intergroup *p* value
PD (mm)																
	T1	18	2.29 ± 0.42	1.95	2.29	2.60	1.40 to 3.00		6	2.26 ± 0.27	1.98	2.29	2.48	1.92 to 2.67		0.882
	T3	18	2.18 ± 0.51	1.64	2.17	2.69	1.33 to 2.92		6	2.11 ± 0.27	1.93	2.08	2.27	1.75 to 2.58		0.756
	T4	18	2.20 ± 0.43	1.83	2.17	2.53	1.67 to 3.08		6	2.37 ± 0.55	1.83	2.29	2.87	1.83 to 3.25		0.503
	T3-T1	18	− 0.11 ± 0.43	-0.43	0.00	0.17	− 1.00 to 0.67	0.347	6	− 0.15 ± 0.35	− 0.42	− 0.20	0.20	− 0.67 to 0.33	0.343	0.732
	T4-T1	18	− 0.08 ± 0.50	-0.58	-0.04	0.41	− 0.75 to 0.75	0.356	6	0.10 to 0.50	− 0.27	0.08	0.47	− 0.59 to 0.92	0.656	0.638
	T4-T3	18	0.02 ± 0.41	-0.25	0.17	0.34	− 0.67 to 0.58	0.737	6	0.26 ± 0.54	− 0.25	0.12	0.83	− 0.25 to 1.08	0.562	0.660
BOP (%)																
	T1	18	0.18 ± 0.18	0.08	0.17	0.27	0.00 to 0.75		6	0.29 ± 0.23	0.06	0.29	0.52	0.00 to 0.58		0.378
	T3	18	0.20 ± 0.17	0.08	0.21	0.33	0.00 to 0.58		6	0.12 ± 0.13	0.00	0.08	0.27	0.00 to 0.33		0.343
	T4	18	0.21 ± 0.20	0.06	0.17	0.42	0.00 to 0.67		6	0.12 ± 0.17	0.00	0.04	0.29	0.00 to 0.42		0.285
	T3-T1	18	0.01 ± 0.22	− 0.11	− 0.04	0.19	− 0.34 to 0.41	0.843	6	− 0.01 ± 0.35	− 0.05	− 0.21	0.14	− 0.50 to 0.33	0.312	0.255
	T4-T1	18	0.02 ± 0.18	− 0.08	0.00	0.19	0.34 to 0.34	0.543	6	− 0.16 ± 0.38	− 0.52	− 0.17	0.14	− 0.58 to 0.34	0.406	0.384
	T4-T3	18	0.01 ± 0.16	− 0.1	0.00	0.17	− .25 to 0.25	0.669	6	0.00 ± 0.19	-0.12	0.00	0.08	− 0.25 to 0.34	0.999	0.783
PCR (%)																
	T1	18	0.19 ± 0.16	0.06	0.17	0.33	0.00 to 0.50		6	0.19 ± 0.21	0.00	0.16	0.42	0.00 to 0.42		0.878
	T3	18	0.10 ± 0.14	0.00	0.08	0.17	0.00 to 0.58		6	0.01 ± 0.03	0.00	0.02	0.02	0.00 to 0.08		0.106
	T4	18	0.11 ± 0.16	0.00	0.00	0.17	0.00 to 0.58		6	0.08 ± 0.20	0.00	0.00	0.12	0.00 to 0.50		0.399
	T3-T1	18	− 0.08 ± 0.18	− 0.27	− 0.08	0.08	− 0.42 to 0.25	0.070	6	− 0.18 ± 0.20	− 0.42	− 0.12	0.00	− 0.42 to 0.00	0.250	0.316
	T4-T1	18	− 0.08 ± 0.23	− 0.27	− 0.08	0.11	− 0.50 to 0.25	0.210	6	− 0.11 ± 0.35	− 0.42	− 0.16	0.12	− 0.42 to 0.50	0.875	0.681
	T4-T3	18	0.00 ± 0.12	− 0.02	0.00	0.11	− 0.25 to 0.17	0.882	6	0.07 ± 0.21	− 0.02	0.00	0.12	− 0.08 to 0.50	0.999	0.919

Negative numbers indicate a decrease and positive numbers indicate an increase. Abbreviations: *PD*, probing depth; *BOP*, bleeding on probing; *PCR*, plaque control record. The differences between the treatment groups (intergroup p-value) were tested with the nonparametric Mann-Whitney *U* test. The intragroup differences over time (paired *p* value) were tested with the nonparametric paired Wilcoxon test

### Aesthetic parameters

Aesthetic outcomes by using the papilla index (Jemt, 1997) and the pink esthetic score (PES) are displayed in Table 2. From prior to tooth preparation to the other time points, the papilla index increased significantly in both groups (*p* < 0.05) indicating an esthetic improvement (Table 2). This gain tended to be superior in the SCTG group (*p* = 0.089). After insertion of the final FDP, both groups remained stable (*p* > 0.05) with a median change of 0 mm at 1 year of follow-up (Table 2). Based on PES analysis, between baseline and one year of follow-up, the median PES values in SCTG group remained stable (from 9.0 to 9.5; *p* = 0.875), while the CONTROL group showed a slight decreased (from 9.5 to 8.00; *p* = 0.007) (Table 2).

**Table 2:** Papilla index for CONTROL and SCTG group prior to tooth preparation (T1), after insertion of the fixed dental prosthesis (T3), and at 1-year follow-up (T4) including the changes between different time-points. Patient-level analysis with means, standard deviations (SD), medians, interquartile ranges (IQR), range from minimum to maximum for both groups (CONTROL and SCTG)

			CONTROL							SCTG						
		*n*	Mean ± SD	Q1	Median	Q3	Range min to max	Paired *p* value	*n*	Mean ± SD	Q1	Median	Q3	Range min to max	Paired *p* value	Intergroup *p* value
Papilla Index																
	T1	18	0.97 ± 0.32	1.00	1.00	1.00	0.00 to 1.50		6	0.42 ± 0.49	0.00	0.25	1.00	0.00 to 1.00		
	T3	18	1.69 ± 0.55	1.00	2.00	2.50	1.00 to 2.50		6	1.67 ± 0.41	1.38	1.75	2.00	1.00 to 2.00		
	T4	18	1.77 ± 0.53	1.25	2.00	2.00	1.00 to 2.50		6	1.67 ± 0.61	1.00	1.75	2.13	1.00 to 2.50		
	T3-T1	18	0.72 ± 0.46	0.38	1.00	1.00	1.00 to 1.50	** < **0.001*	6	1.25 ± 0.61	0.88	1.00	2.00	0.50 to 2.00	0.031*	0.089
	T4-T1	18	0.69 ± 0.60	0.50	1.00	1.00	− 1.00 to 1.50	** < **0.001*	6	1.25 ± 0.69	0.88	1.00	1.75	0.50 to 2.50	0.031*	0.123
	T4-T3	18	− 0.02 ± 0.46	0.00	0.00	0.00	− 1.00 to 1.50	1.000	6	0.00 ± 0.44	− 0.50	0.00	0.5	− 0.50 to 0.50	1.000	0.937
Pink esthetic score																
	T3	18	8.72 ± 3.02	6.50	9.50	11.0	4.0 to 14.00		6	8.33 ± 3.38	6.50	9.00	10.50	2.00 to 12.00		
	T4	18	7.88 ± 3.12	4.50	8.00	13	3.0 to 13.00		6	8.66 ± 3.77	5.25	9.50	11.00	3.00 to 14.00		
	T4-T3	18	− 0.94 ± 1.29	− 1.50	0.00	0.00	− 4.00 to 0.00	0.007*	6	0.33 ± 1.86	− 0.75	0.50	2.00	− 3.00 to 2.00	0.875	0.039*

Negative numbers indicate a decrease and positive numbers indicate an increase. The differences between the treatment groups (intergroup *p* value) were tested with the nonparametric Mann–Whitney *U* test. The intragroup differences over time (paired *p* value) were tested with the nonparametric paired Wilcoxon test. *Represents a statistically significant difference

### Profilometric outcomes

The mean distance (MD) in the buccal pontic area for the different time points and the two groups is displayed in Table 3. The median profilometric contour change amounted to 0.06 mm (prior to tooth preparation to after tooth preparation/insertion of the provisional), 0.02 mm (prior to tooth preparation to post insertion of the final FDP [baseline]) and − 0.25 mm (prior to tooth preparation to one-year after insertion of the final FDP) in the CONTROL group. Conversely, the median profilometric contour changes in the SCTG group amounted to 1.31 mm (prior to tooth preparation to after tooth preparation/insertion of the provisional), 0.96 mm (prior to tooth preparation to post insertion of the final FDP[baseline]), and 0.61 mm (prior to tooth preparation to one-year after insertion of the final FDP). All these profilometric contour changes were significantly more robust in group SCTG (*p* < 0.05) (Table 3). Between baseline (insertion of the final FDP) and one-year of follow-up the median change amounted to − 0.15 mm in the CONTROL group and − 0.48 mm in the SCTG group, with no significance differences between the groups. Positive values indicate a gain in the profilometric contour, whereas negative values indicate a loss in the profilometric contour.

**Table 3:** Clinical outcomes for CONTROL and SCTG prior to tooth preparation (T1), after tooth preparation (T2), after insertion of the fixed dental prosthesis (T3), and at 1-year follow-up (T4) including the changes between different time-points. Patient-level analysis with means, standard deviations (SD), medians, interquartile ranges (IQR), range from minimum to maximum for both groups (CONTROL and SCTG)

			CONTROL							SCTG						
		*n*	Mean ± SD (mm)	Q1	Median (mm)	Q3	Range (mm) min to max	Paired *p* value	*n*	Mean ± SD (mm)	Q1	Median (mm)	Q3	Range (mm) min to max	Paired p-value	Intergroup *p*\ value
MD																
	T1 to T2	18	− 0.14 ± 0.67	− 0.15	0.06	0.14	− 2.01 to 0.77		6	1.25 ± 0.88	0.61	1.31	1.90	− 0.17 to 2.47		0.007*
	T1 to T3	18	− 0.13 ± 0.65	− 0.21	0.02	0.23	− 1.89 to 0.81		6	0.90 ± 0.84	0.42	0.96	1.46	− 0.53 to 2.05		0.011*
	T1 to T4	18	− 0.31 ± 0.73	− 0.36	− 0.25	0.14	− 2.34 to 0.50		6	0.47 ± 0.85	− 0.18	0.61	1.06	− 0.94 to 1.52		0.038*
	T3 to T4	18	− 0.17 ± 0.21	− 0.34	− 0.15	− 0.02	− 0.45 to 0.31		6	− 0.43 ± 0.47	− 0.77	-0.48	− 0.03	− 1.14 to 0.26		0.140
CH																
	T1	18	8.63 ± 1.89	7.95	9.43	9.71	4.14 to 11.00	NA	6	10.28 ± 1.66	8.69	10.40	11.56	8.17 to 12.67	NA	
	T3	18	8.73 ± 1.49	7.97	9.21	9.57	4.37 to 11.16	NA	6	9.11 ± 1.21	7.99	9.41	10.20	7.13 to 10.27	NA	
	T4	18	8.79 ± 1.41	8.15	9.06	9.55	4.36 to 11.06	NA	6	9.15 ± 1.24	8.37	9.23	10.02	7.10 to 10.80	NA	
	T3-T1	18	0.10 ± 0.90	− 0.39	− 0.18	0.44	− 1.32 to 2.32	0.862	6	− 1.17 ± 1.21	− 1.90	− 0.51	− 0.48	− 2.40 to 1.13	0.093	0.011*
	T4-T1	18	0.16 ± 1.09	− 0.58	− 0.22	0.66	− 1.29 to 3.05	0.831	6	− 1.13 ± 1.25	− 2.05	− 1.24	− 1.05	− 2.91 to 0.75	0.094	0.022*
	T4-T3	18	0.05 ± 0.29	-0.13	− 0.03	0.21	− 0.34 to 0.73	0.823	6	0.04 ± 0.45	− 0.41	-0.01	0.54	− 0.51 to 0.62	0.843	0.883
ARW																
	T1	18	8.57 ± 2.58	6.64	7.92	10.49	4.42 to 13.60	NA	6	5.18 ± 1.26	4.06	5.03	6.25	3.83 to 7.05	NA	
	T3	18	8.49 ± 2.26	6.63	8.16	10.13	5.46 to 13.04	NA	6	6.84 ± 1.36	5.63	6.61	8.39	5.19 to 8.52	NA	
	T4	18	8.27 ± 2.19	6.66	8.02	9.70	5.14 to 12.30	NA	6	7.37 ± 1.39	6.35	7.38	8.64	5.20 to 8.98	NA	
	T3-T1	18	-0.07 ± 1.92	-0.55	0.05	1.23	-5.69 to 2.30	0.474	6	1.67 ± 1.64	0.32	1.71	3.05	-0.79 to 3.94	0.093	0.055
	T4-T1	18	-0.30 ± 1.79	-0.74	-0.12	0.70	-5.36 to 1.91	0.772	6	2.19 ± 2.01	0.62	2.23	3.86	-0.78 to 4.85	0.062	0.032*
	T4-T3	18	-0.22 ± 0.64	-0.61	-0.06	0.27	-1.73 to 0.67	0.278	6	0.52 ± 0.91	-0.10	0.20	1.30	-0.40 to 2.10	0.312	0.103

Negative numbers indicate a decrease and positive numbers indicate an increase. Abbreviations: *MD*, mean distance; *CH*, crown height of the pontic; *ARW*, alveolar ridge width. The differences between the treatment groups (intergroup *p* value) were tested with the nonparametric Mann–Whitney *U* test. The intragroup differences over time (paired *p* value) were tested with the nonparametric paired Wilcoxon test. *Represents a statistically significant difference

### Crown height

All data for the crown height (CH) at the pontic site is displayed in Table 3. From prior to tooth preparation to the other time points, the median change of the crown height in the CONTROL group amounted to − 0.18 mm (post insertion of the final FDP) and − 0.22 mm (one-year after insertion of the final FDP), with no significant differences over time (intragroup *p* > 0.05) (Table 3). The median change of the crown height in the SCTG group from prior to tooth preparation to the other time points amounted to − 0.51 mm (post insertion of the final FDP) and − 1.24 mm (one year after insertion of the final FDP) (intragroup *p* > 0.05). After insertion of the final FDP, the crown height remained stable in both groups up to one-year follow-up (Table 3). The median change between the insertion of the final FDP (baseline) and one-year of follow-up amounted to − 0.03 mm in CONTROL group and − 0.01 mm in SCTG group. Negative numbers indicate a coronal displacement of the mid-facial mucosal margin (i.e., a shorter clinical crown height in the pontic area).

### Alveolar ridge width

The alveolar ridge width (ARW) at the pontic sites is displayed in Table 3. From prior to tooth preparation to the other time points, the median change of alveolar ridge width in the CONTROL group amounted to − 0.05 mm (post insertion of the final FDP [baseline]) and − 0.12 mm (1 year after insertion of the final FDP). Conversely, the median change of the alveolar ridge width in the SCTG group from prior to tooth preparation to the other time points amounted to 1.71 mm (post insertion of the final FDP [baseline]) and 2.23 mm (1 year after insertion of the final FDP). Positive numbers indicate an increase in the alveolar ridge width. Expressed differently in the CONTROL group, the median ridge width revealed change of ≈ 3% (prior to tooth preparation to post insertion of the final FDP [baseline]) and 1% (prior to tooth preparation to one-year after insertion of the final FDP). Conversely, the SCTG group demonstrated a median ridge width gain of ≈ 24% (prior to tooth preparation to post insertion of the final FDP [baseline]) and of ≈ 32% (prior to tooth preparation to 1 year after insertion of the final FDP). After insertion of the final FDP, the alveolar ridge remained relatively stable in the CONTROL group with a median loss of − 0.06 mm at one-year of follow-up. The SCTG group, on the other hand, showed a slight increase of 0.2 mm in the alveolar ridge between the insertion of the final FDP and one-year of follow-up.

## Discussion

The present cohort study assessing the pontic site development in sites with or without soft tissue grafting demonstrated (i) a gain in contour and alveolar ridge width due to the surgical procedure (SCTG group) accompanied by stable esthetic outcomes over time; (ii) a slight but continuous reduction of the alveolar ridge in the CONTROL group accompanied by decreased esthetic outcomes; and (iii) stable clinical parameters irrespective of the treatment modality.

Soft tissue augmentation has become a common procedure for pre-prosthetic site development allowing the correction of minor to moderate ridge defects prior to the delivery of conventional tooth-borne fixed dental prostheses, especially at pontic sites [14–16]. The present study revealed a median ridge width gain of ≈ 32% using a SCTG in pontic sites with a follow-up of 1 year. As expected, the major gain occurred before the FDP’s final delivery; nonetheless, it is interesting to note a trend towards a continuous ridge width gain of 0.5 mm (median: 0.2) after the delivery of the final reconstruction. These gain values seem to compare well with those reported in a previous clinical study comparing two different surgical techniques for soft tissue augmentation in single-tooth gaps in the anterior maxilla [14]. In that study, the use of a SCTG resulted in a contour gain of 0.62 mm [14]. Conversely, the CONTROL group in the present study remained rather stable, revealing a minimal reduction of the alveolar ridge width of ≈ 0.3 mm (median: 0.06) at the 1-year follow-up. This minimal—and clinically negligible reduction—might be attributed to the slight compression exerted by the pontic on the soft tissue as previously described ([9, 17].

Adequate site development is pivotal for optimal esthetic outcomes. In this sense, the height of the papillae at the pontic sites can be considered as an important esthetic outcome measure. In general, both groups revealed a significant improvement of the papilla index over time amounting to 1.7 in the CONTROL group and 1.6 in the SCTG group at 1 year of follow-up. According to the JEMT index [12], values close to 2 indicate an acceptable outcome. Despite the similar JEMT score values between the groups at later time points, the improvement in the esthetic outcomes were significantly more stable over time in the SCTG group. This is indicated by the significant reduction in PES values over time found in the control group (mean: from 8.7 to 7.8; median: from 9.5 to 8.0, *p* = 0.007), while in the SCTG group, these PES values remained relatively stable with no significant differences (mean: from 8.3 to 8.6; median: from 9.0 to 9.5, *p* = 0.875). Unfortunately, little attention has been paid to these parameters in FDPs at pontic sites thereby hindering a comparison of the current findings with other studies.

Another critical esthetic parameter is the stability of the soft tissues at the mid-facial mucosal margin at the pontic sites. This stability can be assessed by measuring the possible changes in height of the pontic. Between the insertion of the final FDP and the one-year follow-up, the height of the pontic remained largely unchanged in both groups (changes < 0.05 mm) implying a stable condition of the mid-facial mucosa at pontic sites. These values are inferior to those reported in a previous clinical study comparing the volumetric changes of pontic sites with or without soft tissue grafting [9]. These discrepancies are most likely explained by the shorter follow-up in the present study. While the follow-up in the current study was one year, in the aforementioned study the follow-up was 5 years. It is plausible that the present results might even out at 5 years. A further comparison with other studies could not be performed due to the limited clinical data available.

In order to investigate the early changes at the pontic sites, the intraoral scan data at crown delivery was merged, aligned, and superimposed with the initial situation (prior to tooth preparation). While the control revealed a stability in the crown height and the profilometric buccal contour from prior to preparation to FDP delivery, the SCTG group changed significantly between these time points. In the SCTG group, the height of the virtual pontic decreased by approximately 1 mm. This reduction in the height of the pontic can be explained by the conditioning of the soft tissue by the provisional restoration [18] due to the pressure applied by the provisional. As expected, this pressure also resulted in a reduction of the profilometric buccal contour as observed in the SCTG group. This reduction is most likely related to the quality of the SCTG; in the present study, a single-incision technique was used. Previous reports have indicated that SCTG grafts obtained from the deep palate with this technique might be richer in fatty and glandular tissue [19, 20] than other techniques. The abundance of fatty and glandular tissue in SCTG could lead to graft shrinkage over time. The present findings seem to support those claims and appear to be consistent with previous clinical data showing a decrease in the thickness of the buccal tissue within the first year [21].

Clinical parameters compatible with periodontal health were observed across the groups up to one-year follow-up. This was indicated by the mean PD values around 2 mm in both treatment groups at all time points. The healthy conditions of the periodontal tissues were further supported by the mean values of BOP and PI which remained low in both groups irrespective of the time point. These findings are largely in agreement with one of the few previous reports available on the subject [14].

The present study has several limitations. Firstly, apart from the low sample size, the decision of undergoing soft tissue augmentation relied on clinician’s and patient’s preferences, which is inherently subject to bias. Second, although most of the treated sites had a contour defect at the time of enrolment, the lack of randomization did not allow a balanced distribution of the defects between the groups. Third, the present findings could be limited to the posterior region only, as only posterior areas were treated. In this sense, it is worth noting that the dimensional changes after tooth extraction in the posterior region differs from those in the anterior region [22].

Fourth, in the present study, the amount of keratinized tissue and the clinical attachment level were not evaluated. While the keratinized tissue may have an impact on the esthetic outcomes, the clinical attachment level remains as the gold standard to evaluate is a standard parameter to evaluate regenerative procedures [23] and the stability of the periodontal tissues over time [24]. Fifth, despite the stable esthetic outcomes observed with the use of SCTG, this strategy may still be insufficient to fully restore the buccal convexity, as observed at implant sites [25]. Finally, patient-reported outcomes measures (PROMs) were not assessed. Hence, it remains unclear whether the benefits of applying a SCTG at pontic sites are perceived by the patient. The present findings should be confirmed in future and larger studies.

## Conclusion

Within the limitations of the present study, autogenous soft tissue grafting tends to limit contour changes at pontic sites thus maintaining the esthetic outcomes over time. The lack of soft tissue grafting results in stable clinical outcomes but may affect esthetic outcomes over time.

## Supplementary Information

Below is the link to the electronic supplementary material.

Supplementary file1 (DOCX 3269 KB)
