# Supplementary material for: Pontic site development for fixed dental prostheses with and without soft tissue grafting: 1-year results of a cohort study
Source: Clin Oral Investig. 2022 Jul 1;26(10):6305–16. doi: 10.1007/s00784-022-04582-y (PMC9525322; doi:10.1007/s00784-022-04582-y)
Supplement: Supplementary file 1 — Supplementary file1 (DOCX 3269 KB) [file 784_2022_4582_MOESM1_ESM.docx]

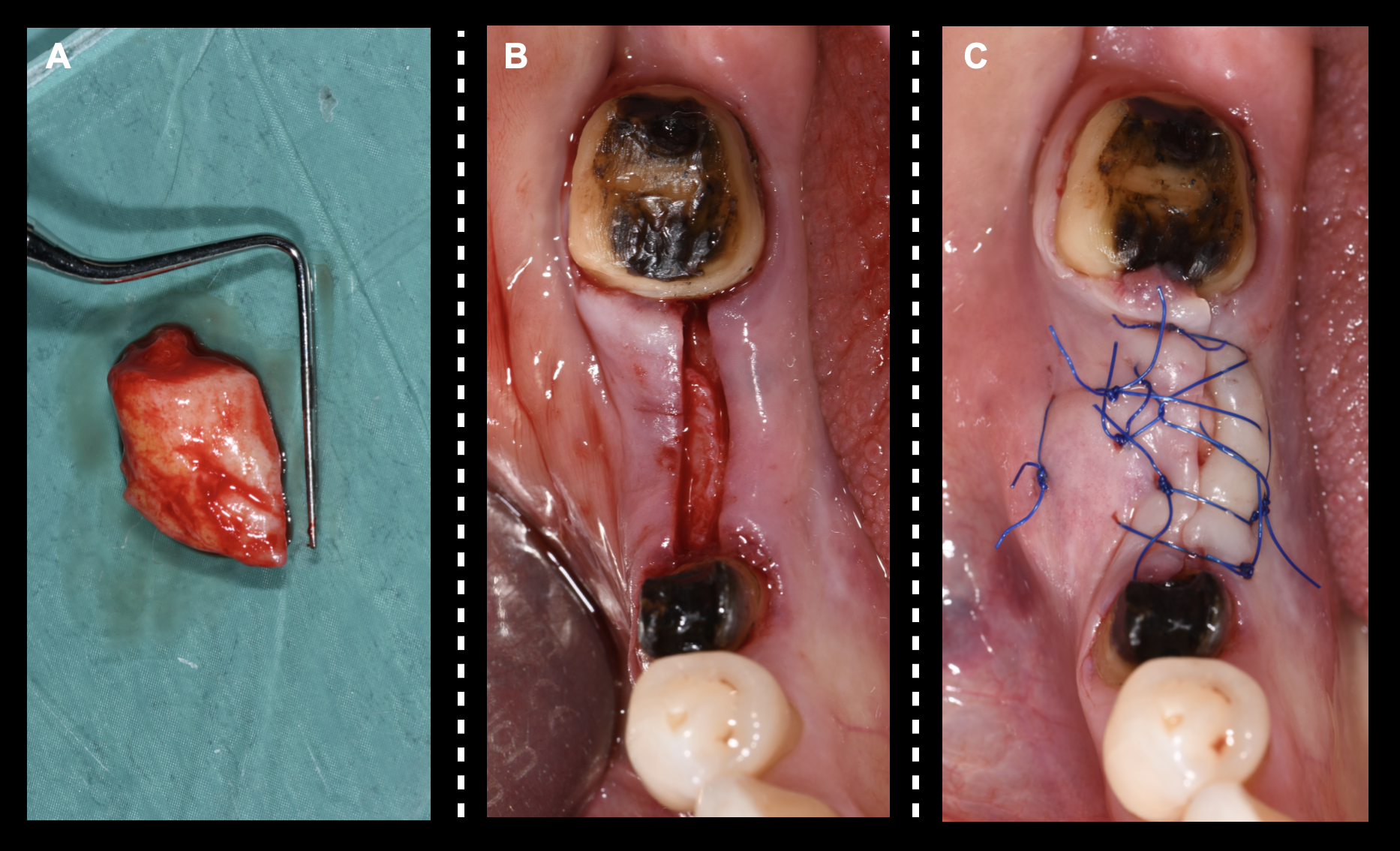


**Supplementary Figure 1.** Autogenous soft tissue grafting. A) SCTG harvested from the palate by single-incision technique. B) SCTG placement in the desired position (buccally and crestally). C) Reposition of the primary flap and wound closure with sutures. Table 1 Clinical outcomes for CONTROL and SCTG group prior to tooth preparation (T1), after insertion of the fixed dental prosthesis (T3), and at 1-year follow-up (T4) including the changes between different time points. Patient-level analysis with means, standard deviations (SD), medians, interquartile ranges (IQR), range from minimum to maximum for both groups (CONTROL and SCTG)
